# *In silico* Assessment of Pharmacological Profile of Low Molecular Weight Oligo-Hydroxyalkanoates

**DOI:** 10.3389/fbioe.2020.584010

**Published:** 2020-11-26

**Authors:** Diana Larisa Roman, Adriana Isvoran, Mǎdǎlina Filip, Vasile Ostafe, Manfred Zinn

**Affiliations:** ^1^Advanced Environmental Research Laboratories, Department of Biology-Chemistry, Faculty of Chemistry, Biology, Geography, West University of Timisoara, Timisoara, Romania; ^2^Institute of Life Technologies, University of Applied Sciences and Arts Western Switzerland (HES-SO Valais-Wallis), Delémont, Switzerland

**Keywords:** oligomers of hydroxyalkanoates, pharmacokinetics profiles, carcinogenicity, cardiotoxicity, endocrine disruption, skin sensitization potency, toxicological endpoints

## Abstract

Polyhydroxyalkanoates (PHAs) are a large class of polyesters that are biosynthesized by microorganisms at large molecular weights (*Mw* > 80 kDa) and have a great potential for medical applications because of their recognized biocompatibility. Among PHAs, poly(3-hydroxybutyrate), poly(4-hydroxybutyrate), poly(3-hydroxyvalerate), poly(4-hydroxyvalerate), and their copolymers are proposed to be used in biomedicine, but only poly(4-hydroxybutyrate) has been certified for medical application. Along with the hydrolysis of these polymers, low molecular weight oligomers are released typically. In this study, we have used a computational approach to assess the absorption, distribution, metabolism, and excretion (ADME)-Tox profiles of low molecular weight oligomers (≤32 units) consisting of 3-hydroxybutyrate, 4-hydroxybutyrate, 3-hydroxyvalerate, 4-hydroxyvalerate, 3-hydroxybutyrate-*co*-3-hydroxyvalerate, and the hypothetical PHA consisting of 4-hydroxybutyrate-*co*-4-hydroxyvalerate. According to our simulations, these oligomers do not show cardiotoxicity, hepatotoxicity, carcinogenicity or mutagenicity, and are neither substrates nor inhibitors of the cytochromes involved in the xenobiotic’s metabolism. They also do not affect the human organic cation transporter 2 (OCT2). However, they are considered to be inhibitors of the organic anion transporters OATP1B1, and OATP1B3. In addition, they may produce eye irritation, and corrosion, skin irritation and have a low antagonistic effect on the androgen receptor.

## Introduction

Polyhydroxyalkanoates (PHAs) are polyesters produced by various microorganisms and serve them as intracellular carbon and energy storage compounds (Zinn et al., 2001). After chemical extraction and purification, PHAs have numerous industrial (e.g., packaging) but also very interesting high value applications in medicine due to their good biocompatibility and biodegradability. Thus, they have been proposed as drug delivery systems (Shrivastav et al., 2013; Radu et al., 2017; Butt et al., 2018; Zhang et al., 2018), implants for bone and cartilage regeneration (Puppi et al., 2010; Ray and Kalia, 2017), tissue engineering matrices (Ray and Kalia, 2017; Butt et al., 2018; Zhang et al., 2018), and anticancer agents (Ray and Kalia, 2017). In 2007, the United States Food and Drug Administration (FDA) approved the first PHA, poly(4-hydroxybutyrate) (P4HB) for use as a surgical suture (Williams et al., 2013).

Other representatives of PHAs with a large potential for biomedical use are poly(3-hydroxybutyrate) (P3HB), poly(3-hydroxyvalerate) (P3HV), poly(4-hydroxyvalerate) (P4HV), as well as their *co*-polymers of 3-hydroxybutyrate (3HB) and 3-hydroxyvalerate (3HV) (P3HB3HV) (Bonartsev et al., 2019), and the hitherto hypothetical poly(4-hydroxybutyrate-*co*-4-hydroxyvalerate (P4HB4HV) (Luef et al., 2015; Ray and Kalia, 2017; Zhang et al., 2018).

It has been found that the chemical property of the monomeric unit(s) and their distribution in the polymer play an important role on the resulting material properties (crystallinity, tensile strength, melting endotherm, etc.). Consequently, degradation speed may vary and is also influenced by the dimensions of the PHA material (e.g., bone implant or nano-sized drug delivery beads). In contrast to poly(L-lactic acid), its degradation process is in addition to hydrolysis also enhanced by enzymatic degradation. As a first degradation product typically oligomeric hydroxyalkanoates (OHAs) and/or monomers are released and many of the latter ones have also been found as natural metabolites in animals (Butt et al., 2018; Zhang et al., 2018; Utsunomia et al., 2020). However, little is known about the interaction of OHAs with the human body and whether the different polymer sizes may result in a particular toxicity.

Consequently, within this simulation study, we focus on the low molecular weight oligomers (containing from 1 to 32 monomeric units) of 3-hydroxybutyrate (O3HB), 3-hydroxyvalerate (O3HV), 4-hydroxybutyrate (O4HB), 4-hydroxyvalerate (O4HV), and *co*-oligomers of 3-hydroxybutyrate and 3-hydroxyvalerate, O(3HB3HV), and the unusual 4-hydroxybutyrate and 4-hydroxyvalerate O(4HB4HV), respectively. By searching in the scientific literature and PubChem database (Kim et al., 2019), we have noticed that, excepting the monomers of 3HB and 4HB, little is known about the biological effects of low molecular weight OHAs, the collected information being presented in Table 1.

**TABLE 1:** State of the art of known biological effects of investigated low molecular weight OHAs.

Oligomer	Observed biological effects
3HB	The oral administration of the 3-hydroxybutyric acid salts increased ketonemia in Wistar rats and conducted to the improvement of body control by the reduction of fat mass and amelioration of serum lipid profile suggesting that ketone supplements could be helpful in the treatment of obesity and metabolic diseases (Caminhotto et al., 2017). Some gastrointestinal disturbances were observed *in vivo* in individuals who consumed a high dose of 3HB (Clarke et al., 2012). A high quantity of 3HB could be harmful because it may conduct to acidosis (Newman and Verdin, 2017). The 3HB may produce eye, skin and respiratory irritations (National Library of Medicine, 2020c).
4HB	The 4HB does not bind to a significant extent to plasma proteins. High doses of 4HB produced hypotension, bradycardia, tachycardia, hypothermia, unconsciousness, acute respiratory acidosis and gastrointestinal disturbances. After ingestion, 4HB is easily absorbed and it is able to cross the blood-brain barrier. It is rapidly metabolized and excreted through the lungs (Busardò and Jones, 2015). The 4HB may produce eye damage, drowsiness or dizziness (National Library of Medicine, 2020a).
3HV	The 3HV may produce eye, skin and respiratory irritations (National Library of Medicine, 2020b).
4HV	The 4HV is marketed as a dietary supplement and replacement for 4HB as at higher doses it shares some effects with 4HB such as sedation, catalepsy, and ataxia (Carter et al., 2005).
O3HB	Low molecular weight O3HB (smaller than 20 monomeric units) are constituents of cells and are covalently attached to proteins located within membranes and organelles (Reusch, 2013).
O3HB, O3HV, O4HB, O4HV, O(3HB3HV)	The *in vitro* cytotoxicity of the OHAs against mouse fibroblasts decreased with increasing OHAs side chain length (Sun et al., 2007).

*3HB, 3-hydroxybutyrate; 3HV, 3-hydroxyvalerate; 4HB, 4-hydroxybutyrate; 4HV, 4-hydroxyvalerate; O3HB, oligomers of 3HB; O3HV, oligomers of 3HV; O4HB, oligomers of 4HB; O4HV, oligomers of 4HV, O(3HB3HV) denotes co-oligomers of 3HB/3HV with various monomeric unit distributions, and O(4HB4HV) denotes co-oligomers of 4HB/4HV with various compositions.*

Thus, in the case of 3HB, it is known that it is a metabolic intermediate produced in the liver from the oxidation of fatty acids released from adipose tissue (Persson, 1970) and is readily consumed in normal mammalian metabolism being an essential carrier of energy and having some cellular signaling functions (Newman and Verdin, 2017). Similarly, 4HB is a natural human metabolite of γ-aminobutyric acid being a neurotransmitter or neuromodulator in the brain. It has a short half-lifetime of about 35 min in the human organism, being rapidly eliminated as exhaled CO_2_ after its metabolic oxidation (Sendelbeck and Girdis, 1985; Maitre, 1997). The presence of 4HB in the human organism has been observed experimentally, trace amounts of 4HB have been found in blood, urine and tissues from healthy individuals that were not exposed to 4HB precursors and/or P4HB (Busardò and Jones, 2015). The chemical 4HB is known as a neurotransmitter and a psychoactive drug, a reason for why it is a controlled substance in the United States since 2000 and in the EU since 2001 (Riviello, 2010).

Taking into account the lack of data concerning the biological effects of the PHA degradation products in general, this study aimed to use a computational approach to predict the Absorption, Distribution, Metabolism, Excretion and Toxicity (ADMET) profiles and possible toxicological endpoints of the low molecular weight O3HB, O4HB, O3HV, O4HV, and their *co*-oligomers.

## Materials and Methods

The oligomers considered in this study were as follows: (i) oligomers of 3-hydroxybutyrate (O3HB) with the number of monomers (u) ranging from 1 to 32; (ii) oligomers of 4-hydroxybutyrate (O4HB, *u* = 1–32); (iii) oligomers of 3-hydroxyvalerate (O3HV, *u* = 1–32); (iv) oligomers of 4-hydroxyvalerate (O4HV, *u* = 1–32). We have also considered *co*-oligomers: 3-hydroxybutyrate-*co*-3-hydroxyvalerate with 2, 3, and 4 monomeric units and various patterns (position of 3HB and 3HV monomers in the oligomeric chain), and 4-hydroxybutyrate-*co*-4-hydroxyvalerate with 2 and 3 monomeric units and various patterns (sequence position of 4HB and 4HV monomers in the chain) of a hypothetical PHA.

**FIGURE 1:**
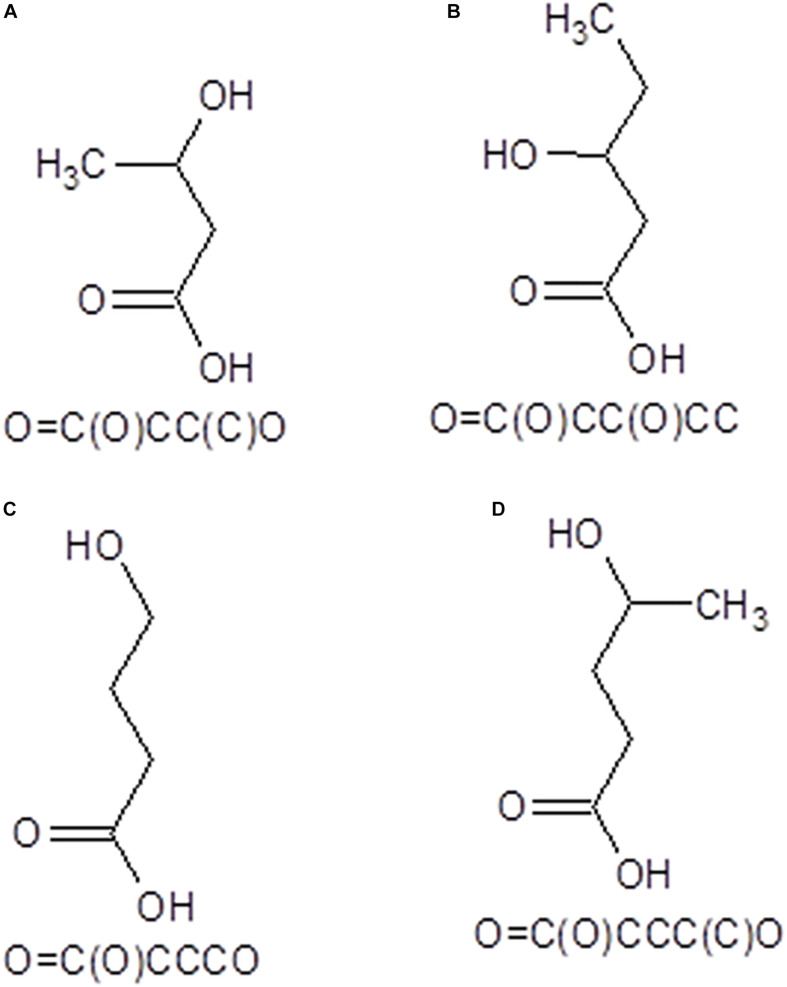
SMILES and 2D structural formulas of the monomeric units of O3HB **(A)**, O3HV **(B)**, O4HB **(C)**, and O4HV **(D)** oligomers that have been investigated in this study.

The Simplified Molecular-Input Line-Entry System (SMILES) formulas and structural *mol* files of the investigated oligomers have been obtained using ACD/ChemSketch (ACD ChemSketch 12.00 and Chemicalize from ChemAxon) utility (https://chemicalize.com accessed in April 2020) and were used further to compute the molecular weights and partition coefficients of these oligomers and to predict their pharmacokinetics profiles as well as their biological effects. The SMILES and two-dimensional formulas of the monomeric units of O3HB, O3HV, O4HB, and O4HV are presented in Figure 1.

Molecular weights and partition coefficients of investigated oligomers have been computed using SwissADME computational facility (Daina et al., 2017)^1^. The partition coefficient between n-octanol and water (logP) is computed in SwissADME by using four freely available predictive models: WLOGP (Wildman and Crippen, 1999), MLOGP (Moriguchi et al., 1992), SILICOS-IT (http://silicos-it.be.s3-website-eu-west-1.amazonaws.com/software/filter-it/1.0.2/filter-it.html, accessed September 2020), and iLOGP (Daina et al., 2014). The consensus value of logP is further computed as the arithmetic mean of the values predicted by the five proposed methods.

Specific literature is abundant and partially integrated incomputational tools for predicting the pharmacokinetic profiles and the biological effects of chemicals. We have selected for this study computational tools that are freely accessible online or are open-source, are developed by recognized groups and benefit of a high number of citations, are based on models with a larger amount of training data, are continuously updated, are robust and their overall accuracy of predictions is usually higher than 70%. These methods have been successfully used for predicting the pharmacological profiles and toxicological endpoints of water-soluble chitosan derivatives (Isvoran et al., 2017), steroids (Roman et al., 2018a), parabens (Roman et al., 2018b), chito-oligomers (Roman et al., 2019), phthalates (Craciun et al., 2019), pesticides (Alves et al., 2018b; Gridan et al., 2019), and low molecular weight oligomers of lactic acid (Dascalu et al., 2020). These methods are summarized in Table 2.

**TABLE 2:** *In silico* tools considered for assessing the ADMET profiles of investigated oligomers.

Computational tool	Predicted activity and accuracy of prediction
admetSAR2.0 is a free available structure-activity relationship database that contains over 210,000 available properties data curated from literature for about 96,000 chemicals. It includes 22 qualitative classification models and 5 quantitative regression models allowing estimations quantitatively described by a probability output (Cheng et al., 2012; Yang et al., 2018). **As input data, this tool uses the SMILES formulas of investigated oligomers and we have extracted the probabilities of ADMET properties**.	It performs predictions concerning: gastrointestinal absorption (GI) (0.965), plasma protein binding (PPB) (0.668), blood brain barrier permeation (BBB) (0.907), substrate/inhibition of the P-glycoprotein (Pgps/Pgpi) (0.802/0.861), substrates (0.779) or inhibitors (0.855) of the human cytochromes (CYPs) involved in the metabolism of xenobiotics, inhibition of organic-anion-transporting polypeptides OATP1B1 (0.886), OATP1B3 (0.927), OATP2B11 (0.885), multidrug and toxin extrusion protein 1 (MATE1) (0.907) and human organic cation transporter OCT2 (0.808), eye corrosion and/or irritation (0.949/0.963), human Ether-a-go-go-Related Gene (hERG) inhibition (0.804), hepatoxicity (0.833), carcinogenicity (0.896), mutagenicity by Ames test (0.843).

ENDOCRINE DISRUPTOME tool uses the molecular docking approach to predict the interactions between the investigated chemicals and 12 human nuclear receptors (Kolsek et al., 2014). **As input data we have used the SMILES formulas of investigated oligomers and extracted data considering the color coded table that was displayed when the molecular docking was finished: green for compounds reflecting a low probability of interacting with the nuclear receptors, orange for compounds revealing a mean probability of interacting with the nuclear receptors and red for compounds revealing a high probability of affecting the nuclear receptors.**	It predicts interactions with (0.780): androgen receptor (AR)—agonistic and antagonistic interactions, estrogen receptors (ER) α and β, glucocorticoid receptor (GR)—agonistic and antagonistic interactions, liver X receptors (LXR) α and β, peroxisome proliferator activated receptors (PPAR) α, β/δ, and γ, retinoid X receptor (RR) α, thyroid receptors (TR) α and β.

Pred-Skin 3.0 is a web-server allowing predictions concerning skin sensitization potential of chemicals based on QSAR models (Braga et al., 2017; Alves et al., 2018a). **SMILES formulas have been used as input data and the probability of oligomers to illustrate skin sensitization or non-sensitization potentials, respectively, the probability maps illustrating the fragments contributions toward skin sensitization potential were retrieved.**	It performs predictions based on five sensitization assay: *in vivo* (murine local lymph node assay, LLNA, accuracy 0.70–0.84), *in chemico* (Direct Peptide Reactivity Assay, DPRA, accuracy 0.73–0.76), *in vitro* (KeratinoSens and human Cell Line Activation Test, H-CLAT, accuracy 0.80–0.86), human repeated insult patch, HRIPT, test and human maximization test, HMT, accuracy 0.70–0.84. There also is a Bayesian consensus model that is generated by averaging the predictions of individual models. Pred-Skin 3.0 also outcomes a probability maps allowing the visualization of the contribution of predicted fragment toward skin sensitization.

Pred-hERG 4.2 is a web tool that builds predictive models of the ability of a chemical compound to inhibit the human Ether-à-go-go Related Gene (hERG) based on the QSAR approach (Braga et al., 2015). **SMILES formulas have been used as input data and the probabilities of oligomers to illustrate hERG K^+^ channel blocking or non-blocking potential, together with the probability maps illustrating the fragments contributions toward hERG blockage potential were retrieved.**	The outcome is a binary prediction of hERG non-blocker or blocker (0.80). This tool also delivers a probability maps allowing the visualization of the contribution of predicted fragment toward hERG blockage.

CarcinoPred-EL (Carcinogenicity Prediction using Ensemble Learning methods) is a computational tool used for predictions concerning the carcinogenicity of chemicals ensemble classification models (Zhang et al., 2017). **SMILES formulas have been used as input data and the software provides a binary prediction (Yes or No) for possible carcinogenicity of the investigated compounds.**	The carcinogenic potential of chemicals is predicted using: Ensemble SVM model (0.691), Ensemble RF model (0.686), Ensemble XGBoost model (0.698).

Toxtree is an open-source application that performs predictions concerning carcinogenicity and mutagenicity by applying a decision tree approach (Patlewicz et al., 2008). **As input data we have used the SMILES formulas of investigated oligomers and we have retrieved the predictions (Yes or No) for the carcinogenic and mutagenic potential of investigated compounds.**	The carcinogenic and mutagenic potential is predicted (0.70).

PASS (Prediction of Activity Spectra of Substances) is a computational tool that predicts biological activity spectra and toxic/side effects starting to the structural formulae of chemical compounds and using the QSAR approach (Poroikov et al., 2007). **This tool also used the SMILES formulas as entry data and provides the probability that the investigated compound to be active for a given adverse biological activity.**	PASS has been used to predict toxic and adverse effects (0.95).

*Numbers in parantheses represent probabilities of predictions.*

For every method, a short description, the predicted biological activity, the accuracy of predictions and references are provided. All these *in silico* tools use as input the SMILES formulas of investigated chemicals. The limits of these tools are mainly expressed by the fact that they do not allow to take into account the dose of the investigated compound. We have used these tools to obtain predictions concerning the ADMET profiles and possible toxicological endpoints of the considered oligomers. We must mention that there is no consideration concerning the chirality of the investigated compounds.

Usually, these predictions are expressed as probabilities for a given compound to have (positive values) or not to have (negative values) a tested biological activity. We have considered that a predicted biological effect has a chance to be detected experimentally if the computed probability is higher than 0.7. This threshold has been considered because in this case the investigated compound reveals a high similarity to compounds with known biological activity and used for building the statistical model (Filimonov et al., 2014). The outcomes of the used computational tools are compared with each other, the correlation/un-correlations are emphasized, and all the results are discussed taking into account the information available in scientific publications and the limitations of the *in silico* tools.

## Results

The molecular weights and the consensus values of partition coefficients for the studied oligomers, computed using SwissADME tool, are given in Table 3.

**TABLE 3:** Molecular weight (MW) and lipophilicity (logP) of oligomers considered in this study.

Oligomer	MW (g/mol)	log*P*	Oligomer	MW (g/mol)	log*P*
O3HB 1u	104.10	−0.19	O4HB 1u	104.10	−0.16
O3HB 2u	190.19	0.33	O4HB 2u	190.19	0.39
O3HB 3u	276.28	0.78	O4HB 3u	276.28	0.77
O3HB 4u	362.37	1.17	O4HB 4u	362.37	1.13
O3HB 5u	448.46	1.53	O4HB 5u	448.46	1.67
O3HB 6u	534.55	1.93	O4HB 6u	534.55	2.09
O3HB 7u	620.64	2.25	O4HB 7u	620.64	2.49
O3HB 8u	706.73	2.62	O4HB 8u	706.73	2.86
O3HB 16u	1395.44	5.29	O4HB 16u	1395.44	5.98
O3HB 20u	1739.80	6.92	O4HB 20u	1739.80	7.47
O3HB 24u	2084.16	8.13	O4HB 24u	2084.16	9.27
O3HB 28u	2428.51	6.75	O4HB 28u	2428.51	7.29
O3HB 32u	2772.87	7.72	O4HB 32u	2772.87	8.34
O3HV 1u	118.13	0.12	O4HV 1u	118.13	0.17
O3HV 2u	218.25	1.05	O4HV 2u	218.25	0.99
O3HV 3u	318.36	1.82	O4HV 3u	318.36	1.68
O3HV 4u	418.48	2.53	O4HV 4u	418.48	2.44
O3HV 8u	818.94	5.46	O4HV 8u	818.94	5.38
O3HV 12u	1219.41	8.26	O4HV 12u	1219.41	8.14
O3HV 16u	1619.87	10.79	O4HV 16u	1619.87	10.68
O3HV 20u	2020.33	13.74	O4HV 20u	2020.33	13.24
O3HV 24u	2420.79	16.84	O4HV 24u	2420.79	16.60
O3HV 28u	2821.26	14.96	O4HV 28u	2821.26	14.01
O3HV 32u	3221.72	17.07	O4HV 32u	3221.72	15.98
O3HVB	204.22	0.69	O4HBV	204.22	0.67
O3HBV	204.22	0.71	O4HVB	204.22	0.69
O3HVBV	304.34	1.47	O4HBVB	290.31	1.14
O3HBVB	290.31	1.09	O4HBVV	304.34	1.42
O3HVBVB	390.43	1.89	04HVBV	304.34	1.38
O3HBVBV	390.43	1.86	O4HVBB	290.31	1.09

The oligomers considered in the present study have a molecular weight between 104.1 and 3221.72 g/mol (see Table 3). The values of partition coefficients are slightly distinct for oligomers containing the same number of monomers such as 3-hydroxybutyrate and 4-hydroxybutyrate, and 3-hydroxyvalerate and 4-hydroxyvalerate, respectively. This is also true for *co*-oligomers of 3-hydroxybutyrate-*co*-3-hydroxyvalerate and 4-hydroxybutyrate-*co*-4-hydroxyvalerate containing the same number of monomeric units but with different sequence patterns. As the partition coefficient is an important physicochemical property for pharmacokinetics of xenobiotics, it underlines the significance of the pattern in the case of *co*-oligomers for their biological interactions.

**FIGURE 2:**
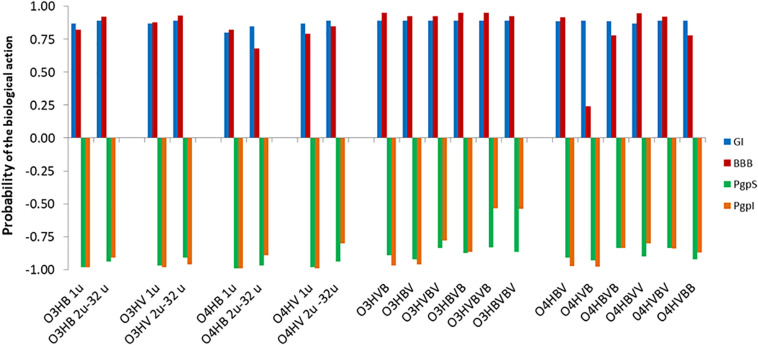
Absorption profiles of low molecular weight oligo-hydroxyalkanoates calculated with admetSAR2.0: u denotes the number of units in the oligomer, O3HB denotes the oligomer of 3HB, O3HV denotes the oligomer of 3HV, O4HB denotes the oligomer of 4HB, O4HV denotes the oligomer of 4HV. In the case of *co*-oligomers, BV, VB, BVB, VBV, BVV, VBB, BVBV, and VBVB, respectively, illustrate the succession of the 3-hydroxybutyrate/4-hydroxybutyrate (B) and 3-hydroxyvalerate/4-hydroxyvalerate (V) monomers in the *co*-oligomeric chain, respectively. The predicted probabilities for gastrointestinal absorption (GI), for penetration of the blood-brain barrier (BBB), or to be a substrate or an inhibitor pf P-glycoprotein (PgpS/PgpI) take values between 0 and 1 in the case of a biological activity that is present and between −1 and 0 when the activity is considered absent. For values closer to 1, the biological effects are highly probable and values closer to −1 correspond to highly improbable biological effects.

**FIGURE 3:**
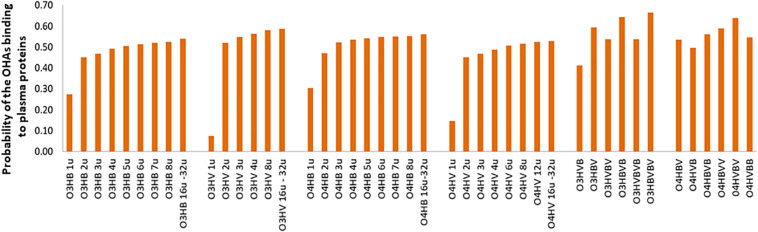
Probabilities of the OHAs binding to plasma proteins (PPB) of low molecular weight oligo-hydroxyalkanoates calculated by admetSAR2.0. Abbreviations are used as explained in Figure 2.

Concerning the absorption and distribution profiles, admetSAR2.0 tool calculates for every investigated oligomer the probabilities to reveal gastrointestinal absorption (GI), to be able to penetrate the blood-brain barrier (BBB), to be a substrate or an inhibitor of P-glycoprotein (PgpS/PgpI) (Figure 2), and to be able to bind to plasma proteins (PPB) (Figure 3). The predicted probabilities take values between 0 and 1 when the investigated biological activity is considered to be present and between −1 and 0 when the biological activity is considered being absent. A probability value closer to 1 or −1 indicates a biological effect that is highly probable or highly improbable, respectively.

Figure 2 suggests that there are high probabilities of gastrointestinal absorption of all OHAs, the capability of all investigated OHAs to penetrate the blood-brain barrier and that these compounds should be considered neither substrates nor inhibitors for the P-glycoprotein (P-gp).

Figure 3 advocates a reduced potential of the investigated OHAs to bind to plasma proteins (PPB) as all values of the probabilities are lower than 0.7. This potential moderately increases with the molecular weight. *Co*-oligomers illustrate higher probabilities to bind to plasma proteins, and *co*-oligomers containing the same number of monomers but with different sequence patterns reveal distinct probabilities to bind to plasma proteins. It emphasizes the important role of the molecular weight and oligomer’s pattern on the biological activity of investigated OHAs.

Regarding the effects of the investigated OHAs on the metabolism of endogenous and exogenous compounds, admetSAR2.0 tool suggests that none of the investigated oligomers has to be considered as a substrate for the human cytochromes involved in the metabolism of xenobiotics. Also, they are not able to inhibit these cytochromes (Supplementary Table 1).

All investigated OHAs revealed high probabilities of inhibition of the organic-anion-transporting polypeptides (OATP) 1B1 (OATP1B1) and 1B3 (OATP1B3) and do not affect the multidrug and toxin extrusion protein 1 (MATE1), OATP2B1 and the human organic cation transporter 2 (OCT2) (Table 4).

**TABLE 4:** Probabilities of inhibition of organic anion and cation transporting polypeptides by the low molecular weight OHAs: OATP, organic anion transporting polypeptide; OCT, organic cation transporting polypeptide; MATE1, multidrug and toxin extrusion protein 1.

Oligomer	OATP2B1i	OATP1B1i	OATP1B3i	MATE1i	OCT2i
O3HB 1u	–0.87	0.96	0.96	–1.00	–0.98
O3HB 2u	–0.85	0.93	0.96	–0.98	–1.00
O3HB 3u–8u	–0.85	0.93	0.95	–0.98	–0.98
O3HB 16u–32u	–0.71	0.93	0.95	–0.98	–0.98
O4HB 1u	–0.85	0.93	0.95	–1.00	–0.90
O4HB 2u	–0.84	0.92	0.96	–0.96	–0.83
O4HB 3u–8u	–0.85	0.92	0.96	–0.96	–0.83
O4HB 16u–32u	–0.86	0.92	0.96	–0.96	–0.83
O3HV 1u	–0.84	0.94	0.96	–1.00	–0.95
O3HV 2u–7u	–0.85	0.90	0.96	–0.98	–0.90
O3HV 8U	–0.71	0.90	0.95	–0.98	–0.88
O3HV 16u–32u	–0.57	0.90	0.95	–0.98	–0.88
O4HV 1u	–0.84	0.95	0.95	–1.00	–0.93
O4HV 2u–5u	–0.85	0.93	0.96	–0.98	–0.95
O4HV 6u–32u	–0.57	0.93	0.95	–0.98	–0.95
O3HVB	–0.85	0.89	0.95	–0.98	–0.90
O3HBV	–0.85	0.90	0.96	–0.98	–0.93
O3HVBV	–0.85	0.89	0.94	–0.98	–0.90
O3HBVB	–0.85	0.88	0.95	–0.98	–0.88
O3HVBVB	–0.85	0.88	0.95	–0.98	–0.88
O3HBVBV	–0.85	0.90	0.94	–0.98	–0.90
O4HBV	–0.84	0.93	0.95	–0.92	–0.78
O4HVB	–0.84	0.91	0.96	–0.98	–0.90
O4HBVB	–0.85	0.90	0.94	–0.98	–0.83
O4HBVV	–0.85	0.92	0.95	–0.98	–0.78
04HVBV	–0.85	0.92	0.96	–0.98	–0.85
O4HVBB	–0.85	0.92	0.96	–0.98	–0.90

*Abbreviations are used as explained in Figure 2.*

Predictions obtained using admetSAR2.0 concerning toxicological endpoints of investigated OHAs suggest that they may not be considered neither as carcinogens not mutagens are not potential inhibitors of the hERG K^+^ channel, and do not induce hepatotoxicity. However, they illustrate a moderate potential of producing eye corrosion and oligomers having low molecular weights may also produce eye irritations (Supplementary Table 2). We must mention that none of the hypothetical *co*-oligomers containing 4HB and 4HV that were part of this study have been predicted to cause eye irritation. The ability of the monomers 3HB, 3HV, 4HB, and 4HV to trigger eye, skin, and respiratory irritations has also been forecast using the PASS tool (see below) and few literature data are confirming these effects for 3HB, 3HV, and 4HB (see Table 1).

**FIGURE 4:**
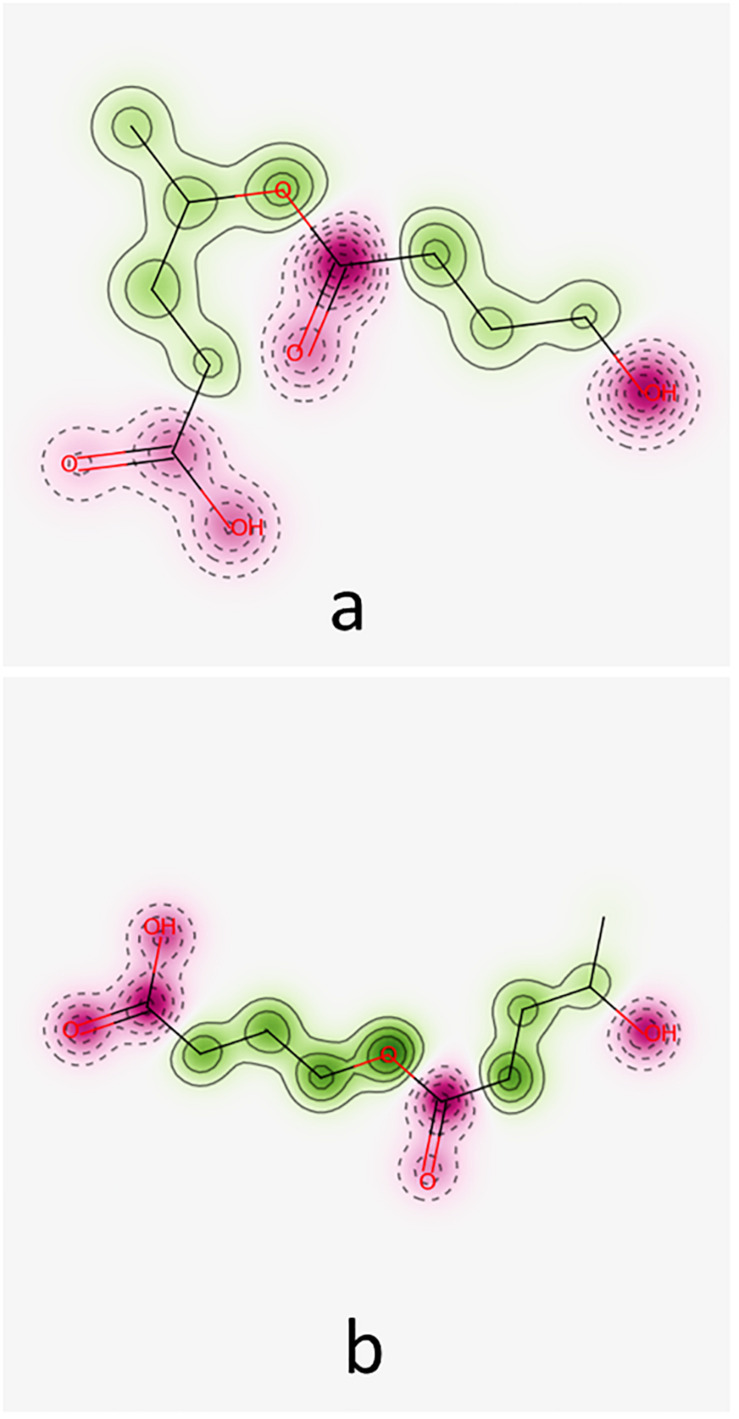
Probability maps illustrating the contribution of fragments of *co*-oligomers O4HBV **(a)** and O4HVB **(b)** toward hERG K^+^ channel blockage. Fragments in green represent contributions toward blockage of hERG, pink fragments contribute to a decrease of hERG blockage.

Predictions obtained by using Pred-hERG 4.2 are illustrated in Figure 4 for the hypothetical *co*-oligomers O4HBVV and O4HVBB. This Figure 4 emphasizes the probability map illustrating the contribution of fragments (green regions) of the *co*-oligomers to produce hERG K^+^ channel blockage.

The *co*-oligomers O4HBVV and O4HVBB contain fragments contributing to both increasing and decreasing hERG K^+^ channel blockage. Synthesis of all results furnished by Pred-hERG 4.2 tool is presented in Table 5, suggesting that these compounds do not own a hERG blockage potential. Table 5 also contains the synthesis of the results regarding the skin sensitization potential (see below).

**TABLE 5:** Predictions concerning cardiotoxicity obtained using Pred-hERG 4.2 computational tool and concerning skin sensitization potential obtained using the Bayesian consensus model under Pred-Skin3.0 tool for the investigated oligomers.

Oligomer/Properties	Cardiotoxicity potency (confidence)	Skin sensitization potential (Bayesian outcome)
O3HB 1U	Non-cardiotoxic (1)	Non-sensitizer
O3HB 2U	Non-cardiotoxic (0.9)	Non-sensitizer
O3HB 3U	Non-cardiotoxic (0.8)	Non-sensitizer
O3HB 4U–8U	Non-cardiotoxic (0.7)	Non-sensitizer
O3HB 16U–32U	Too big to be computed	Non-sensitizer
O4HB 1U	Non-cardiotoxic (0.9)	Too big to be computed
O4HB 2U–20U	Non-cardiotoxic (0.7)	Non-sensitizer
O4HB 24U–32U	Too big to be computed	Non-sensitizer
O3HV 1U	Non-cardiotoxic (0.9)	Non-sensitizer
O3HV 2U	Non-cardiotoxic (0.8)	Non-sensitizer
O3HV 3U	Non-cardiotoxic (0.7)	Too big to be computed
O3HV 4U–8U	Non-cardiotoxic (0.6)	Non-sensitizer
O3HV 16U–32U	Too big to be computed	Non-sensitizer
O4HV 1U	Non-cardiotoxic (0.9)	Non-sensitizer
O4HV 2U	Non-cardiotoxic (0.9)	Non-sensitizer
O4HV 3U–16U	Non-cardiotoxic (0.6)	Non-sensitizer
O4HV 20U–32U	Too big to be computed	Too big to be computed
O3HVB, O3HBV	Non-cardiotoxic (0.8)	Non-sensitizer
O3HVBV, O3HBVB	Non-cardiotoxic (0.7)	Non-sensitizer
O3HVBVB, O3HBVBV	Non-cardiotoxic (0.6)	Non-sensitizer
O4HBV	Non-cardiotoxic (0.8)	Non-sensitizer
O4HVB	Non-cardiotoxic (0.7)	Too big to be computed
O4HBVB, O4HBVV, O4HVBV, O4HVBB	Non-cardiotoxic (0.6)	Non-sensitizer

*The confidence of predictions obtained using Pred-hERG4.2 is also illustrated. Abbreviations are used as explained in Figure 2.*

**FIGURE 5:**
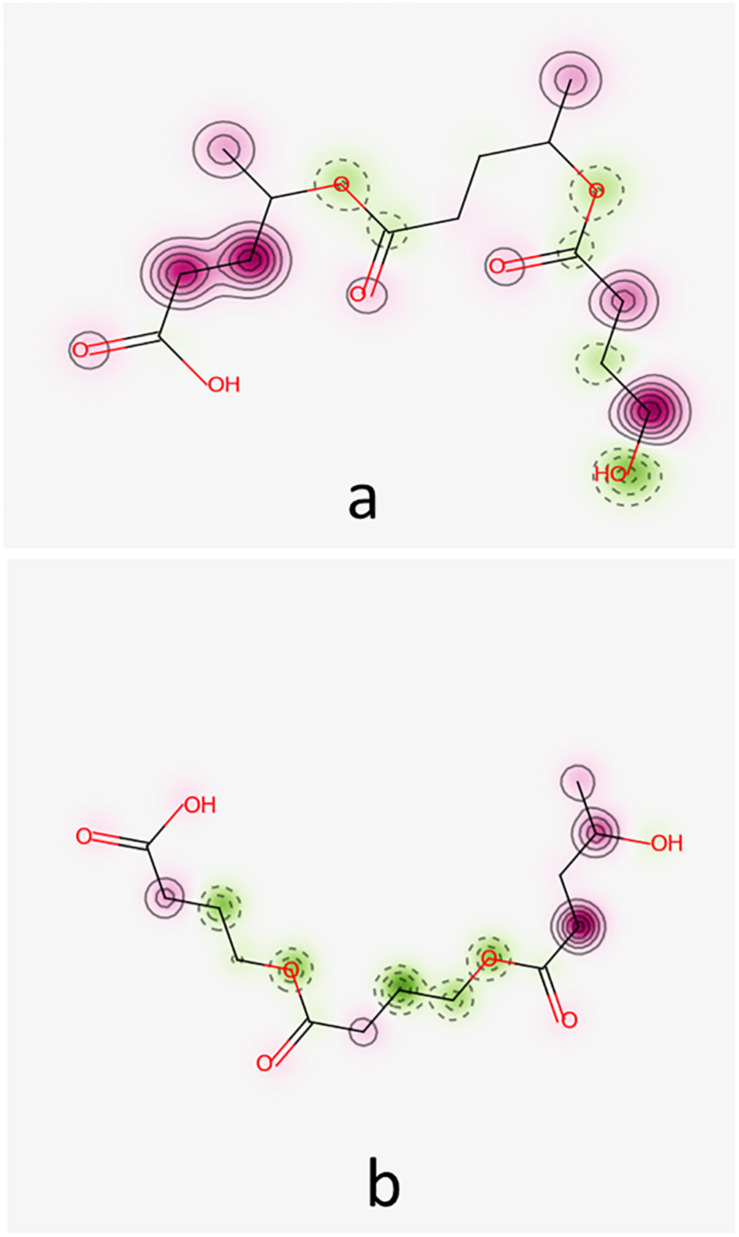
Probability maps illustrating the contribution of fragments of *co*-oligomers O4HBVV **(a)** and O4HVBB **(b)** to skin sensitization potential based on KeratinoSens model. Fragments in green illustrate an increase in skin sensitization potential, the pink fragments contribute to decrease of skin sensitization.

Pred-Skin 3.0 computational tool has been used to obtain prediction concerning the skin sensitization potential of the investigated oligomers. Figure 5 illustrates the probability maps reflecting the contribution of fragments (pink regions) of the hypothetical *co*-oligomers O4HBVV and O4HVBB on the skin sensitization potential based on the KeratinoSens model.

Figure 5 reveals that O4HBVV contains fragments that may contribute to decreasing the skin sensitization potential, but O4HVBB contains fragments suggesting a skin sensitization potential. This is in good agreement with the consensus model, as presented in Table 5. Information furnished by Pred-Skin3.0 online tool by using the five models based on skin sensitization assays is presented in Supplementary Table 3.

Predictions obtained using CarcinoPred-El and Toxtree (Supplementary Table 4) are in good correlation with each other and with those obtained using admetSAR2.0, advocating that investigated OHAs should not be considered as reflecting carcinogenicity nor mutagenicity.

ENDOCRINE DISRUPTOME computational tool suggested that investigated OHAs have only a weak potential to produce an antagonistic effect on the androgen receptor (AR) and oligomers containing 6–8 monomeric units may also weakly affect the glucocorticoid receptor (GR) (Supplementary Table 5). We also underline that oligomers with higher molecular weight (more than 8 monomeric units) were too big to accommodate in the binding cavities of considered nuclear receptors and therefore calculations have been aborted.

The predicted side effects of OHAs were obtained using the PASS tool and are listed in Table 6.

**TABLE 6:** Predicted side effects of the low molecular weight OHAs using PASS software.

Oligomer	Predicted side effects
O3HB 1U	Toxic by respiration (0.968), metabolic acidosis (0.927), eye irritation (0.907).
O3HB 2U–12U	Toxic by respiration (0.971), eye irritation (0.957).
O4HB 1U	Toxic by respiration (0.968), acidosis metabolic (0.948), euphoria (0.948), skin irritation (0.940)
O4HB 2U–12U	Toxic by respiration (0.979), euphoria (0.951), acidosis, metabolic (0.949), weakness (0.937), muscle weakness (0.935), eye irritation (0.929), conjunctivitis (0.915), dyspnea (0.905).
O3HV 1U	Toxic by respiration (0.944).
O3HV 2U–12U	Eye irritation (0.964), skin irritation (0.919), toxic by respiration (0.912), conjunctivitis (0.906).
O4HV 1u	Toxic by respiration (0.981), Acidosis metabolic (0.933), eye irritation (0.921), skin irritation (0.906).
O4HV 2U–12U	Toxic by respiration (0.982), eye irritation (0.963), skin irritation (0.918).
O3HVB	Eye irritation (0.975), skin irritation (0.940), toxic by respiration (0.926), conjunctivitis (0.912).
O3HBV, O3HVBV, O3HBVB, O3HVBVB, O3HBVBV	Eye irritation (0.978), skin irritation (0.964), toxic by respiration (0.944).
O4HBV, O4HVB, O4HBVB, O4HBVV, 04HVBV, O4HVBB	Eye irritation (0.982), toxic by respiration (0.980), skin irritation (0.973), conjunctivitis (0.925), dyspnea (0.919), acidosis metabolic (0.914).

*The numbers in parentheses represent the probabilities that the mentioned side effects will occur. The predicted probabilities take values between 0 and 1. The values closer to 1 illustrate a high probability that these oligomers produce the mentioned side effects. Abbreviations are used as explained in Figure 2.*

There are some side effects predicted for all investigated OHAs: toxicity by respiration, eye irritation, skin irritation, and conjunctivitis.

## Discussion

The high probabilities of gastrointestinal absorption of OHAs that are illustrated in Figure 2 correspond with known data indicating the rapid absorption of hydroxyalkanoates (Busardò and Jones, 2015; Caminhotto et al., 2017) and of 3HB (Cuenoud et al., 2020), the blood-brain permeability for ketone bodies, and the use of 4HB as neurotransmitter and psychoactive drug (National Library of Medicine, 2020a). Figure 2 also suggests that the analyzed compounds are neither substrates nor inhibitors for the P-glycoprotein (P-gp). This protein is expressed in various normal tissues and has a protective role against xenobiotic substances including toxins (Cascorbi, 2011). Our results suggest that the presence of OHAs in the human organism does not alter pharmacokinetics of drugs by being substrates and/or by inhibiting the P-gp.

There are some interesting differences between the predicted pharmacokinetics of oligomers and *co*-oligomers. Thus, gastrointestinal absorption and the potential of blood-brain barrier penetration are predicted to be higher for the *co*-oligomers. Moreover, there are small differences between the predicted probabilities of GI, BBB, PPB, and PgpS/PgpI for *co*-oligomers containing the same number of monomeric units but having distinct sequence patterns.

The ability of OHAs to bind to plasma proteins has been predicted to be low. However, it was found that the probability to bind to plasma proteins gradually increases with the molecular weight. The *co*-oligomers containing the sequences 3-hydroxybutyrate-3-hydroxyvalerate or 4-hydroxybutyrate-4-hydroxyvalerate revealed to have the highest probabilities to bind to plasma proteins. Low probabilities to bind to plasma proteins have been also predicted for low molecular weight chitosan based oligomers (Roman et al., 2019) and low molecular weight oligomers of lactic acid (Dascalu et al., 2020). This outcome is also in good agreement with the information that the binding of 4HB to plasma proteins is negligible and its distribution on the human organism depends on its distribution in the total body water (Busardò and Jones, 2015).

The human OATPs and OCTs handle a variety of endogenous and xenobiotic substrates playing a significant role in the absorption, distribution, and elimination of chemicals. OATP1B1, OATP1B3, OATP2B1, MATE1, and OCT2 are considered when investigating the interactions of chemicals with transporter polypeptides. This is of great importance because diverse classes of drugs are known as substrates of these transporters and knowledge about inhibition of these transporters is of great value in revealing actions in complex systems like hepatocytes (Karlgren et al., 2012; Belzer et al., 2013; Wittwer et al., 2013). Furthermore, inhibition of any of these transporters may result in potential drug-xenobiotics interactions, alterations in chemicals exposure and their accumulation in various tissues. The existence of high-quality data concerning the inhibition of these transporters allowed statistical modeling and, consequently, they are considered by numerous computational tools. OATP1B 1 and OATP1B3 are mainly expressed in the human liver (König et al., 2000) and are responsible for the uptake of bile acids and a broad range of other organic anions being involved in the hepatic clearance (Geier et al., 2007; Alam et al., 2018). OATP2B1 is a transporter with a narrow substrate specificity and is expressed in various human tissues including the liver, kidney, lung, and small intestine (Karlgren et al., 2012). MATE1 is widely expressed in the human body tissues and contributes to the tissue distribution and excretion of many drugs (Wittwer et al., 2013). OCT2 facilitates the renal secretion of organic cations being considered as a potential target for undesirable drug-drug interactions and numerous studies envisaged the understanding of the basis of its selectivity (Belzer et al., 2013). Data presented in Table 4 suggest that all investigated OHAs are potential inhibitors of OATP1B1 and OATP1B3. Decreased transport function of OATP1B1 and OATP1B3 may lead to drug-drug interactions and severe adverse events. Consequently, the US Food and Drug Administration recommended evaluating the inhibitory potential of new chemicals against OATP. It underlines the importance of predictions concerning the inhibition of OATPs by OHAs.

Data presented in Table 5 suggest the non-blocking potential of every of the investigated oligomers toward hERG K^+^ channel and are in very good agreement with predictions obtained using admetSAR2.0 computational facility. Table 5 also advocates that, excepting the *co*-oligomers O3HBVBV, O4HVB, and O4HVBB, the other investigated oligomers do not show skin sensitization potential. This result underlines that *co*-oligomers have biological activities that are different compared with the ones found for homo-oligomers and that the biological effects also depend on the composition of the *co*-polymer with respect to monomeric units. This result is in good correlation with literature data presented by Bonartsev et al. (2019) and revealing that *co*-oligomers have distinct properties and actions than homo-oligomers (Bonartsev et al., 2019).

Predictions presented in Table 6 are in good agreement with specific literature data (see Table 1). The side effects predicted for all investigated OHAs, i.e., toxic by respiration, eye irritation, skin irritation, may be important especially for professional exposure in case of powders. Furthermore, the results of this study highlight that *co*-oligomers show quite distinct biological effects compared with those of homo-oligomers and that the biological effects usually depend on the positions of HB and HV monomers in the chain.

Quite similar results with those emphasized in this study were obtained when assessing the pharmacological profiles of the short oligomers resulting from the degradation of other biopolymers that are used for medical applications: oligomers (up to 40 monomeric units) of lactic acid (OLAs) (Dascalu et al., 2020) and low molecular weight chito-oligosaccharides (Roman et al., 2019). OLAs were also predicted as being able to inhibit the organic anion transporting peptides OATP1B1 and/or OATP1B3. In addition, they showed a minor probability of affecting the androgen and glucocorticoid receptors but their powders may produce eye injuries. Chito-oligosaccharides containing up to 8 monomeric units (glucosamine and/or N-acetyl glucosamine) were also suggested as potential inhibitors of the organic anion transporting polypeptides 1B1 and 1B3. They illustrated a minor probability of affecting the androgen receptor and they are considered to be able to produce cardiotoxicity and phospholipidosis.

The results of this study, and those of similar studies published in scientific literature, emphasize the importance of assessment of the physiological effects of PHAs and of their degradation products in the human organism and, in general, the necessity of designing novel polymers for medical applications with tailor-made properties (e.g., P4HB4HV). The limitations of our study are the disregard of the chirality of the monomeric units of OHAs and of the concentration of investigated compounds when the computational predictions of ADMET profiles were realized.

## Conclusion

Low molecular weight oligo-hydroxyalkanoates may result from the degradation of polyhydroxyalkanoate based implants or medical devices (e.g., drug delivery systems). Due to their presence in the human organism, it is necessary to investigate their pharmacokinetics profiles and to identify possible toxicological effects at a very early stage of research. The present study suggests favorable pharmacokinetic profiles for all types of OHAs. The investigated oligomers are not considered to reflect any hepatotoxicity, cardiotoxicity, mutagenicity nor carcinogenicity. There are a few predicted side effects for the investigated OHAs: the potential to produce eye, skin and respiratory irritation (in the case of exposure to their powders), antagonistic effects against the androgen receptor and inhibition of the OATP1B1 and OATP1B3. Few *co*-oligomers (O3HBVBV, O4HVB, O4HVBB) revealed high probabilities to produce a skin sensitization potential. The effects of eye, skin and respiratory irritations, and skin sensitization potential must be taken into consideration especially for people who are professionally exposed (e.g., during production or packaging of these polymers).

In our study, we could not find any significant differences between the predicted effects for distinct types of OHAs: O3HB, O4HB, O3HV, O4HV. There are dissimilarities in the predicted probabilities reflecting pharmacokinetics properties and side effects for oligomers by comparison to *co*-oligomers and for *co*-oligomers containing the same number of monomeric units but having distinct sequence patterns.

There is a good agreement between the predictions obtained using various computational tools and concerning the biological/toxicological effects that have been investigated. Furthermore, there is a good correlation between the obtained predictions and literature experimental data, when available (which is not the case for the hypothetical P4HB4HV).

The results presented in this study suggest that the therapeutic use of PHAs offers new opportunities at moderate risks. Moreover, these results could be used to define or to guide *in vitro* and *in vivo* toxicity tests such as to contribute to the selection of best prototypes for medical applications. Implementation of defined *co*-polymeric structures, such as the *co*-polymer consisting of 4HB/4HV, could conduct to the further optimization of the properties of the biomaterial.

## Data Availability Statement

All datasets generated for this study are included in the article/Supplementary Material, further inquiries can be directed to the corresponding author.

## Author Contributions

DR performed the computations using Pred-Skin3.0, Pred-hERG4.2, CarcinoPredEL, Toxtree, and Endocrine Disruptome, and contributed to results analysis and manuscript editing. MF performed computations using admetSAR2.0 and PASS online and contributed to results analysis. VO furnished the SMILES and structural formulas of oligomers and contributed to the conception and design of the manuscript. AI and MZ conceived and planned the study and contributed to the analysis and conception of the results, design and editing of the manuscript. All authors contributed to the article and approved the submitted version.

## Conflict of Interest

The authors declare that the research was conducted in the absence of any commercial or financial relationships that could be construed as a potential conflict of interest.

## Footnotes

1 http://www.swissadme.ch/
