# Supplementary material for: In silico Assessment of Pharmacological Profile of Low Molecular Weight Oligo-Hydroxyalkanoates
Source: Front Bioeng Biotechnol. 2020 Nov 26;8:584010. doi: 10.3389/fbioe.2020.584010 (PMC7726197; doi:10.3389/fbioe.2020.584010)
Supplement: Supplementary file 1 [file Table_1.DOCX]

Supplementary Table 1. Predictions obtained using admetSAR2.0 tool concerning the probability that low molecular weight oligo-hydroxyalkanoates are substrates and inhibit the human cytochromes involved in the metabolism of xenobiotics. The predicted probabilities take values between 0 and 1 in the case of a biological activity that is present and between -1 and 0 when the activity is considered absent. Values closer to 1 indicate that the biological effects are highly probable and values closer to -1 correspond to highly improbable biological effects. In this table u denotes the number of units in the oligomer, O3HB denotes the oligomer of 3HB, O3HV denotes the oligomer of 3HV, O4HB denote the oligomer of 4HB, O4HV denotes the oligomer of 4HV. In the case of co-oligomers, BV, VB, BVB, VBV, BVV, VBB, BVBV and VBVB illustrate the succession of the butyrate (B) and respectively valerate (V) monomers in the oligomer chain.

| **Oligomer** | **CYP3A4s** | **CYP2C9s** | **CYPSD6s** | **CYP3A4i** | **CYP2C9i** | **CYP2C19i** | **CYP2D6i** | **CYP1A2i** |
| --- | --- | --- | --- | --- | --- | --- | --- | --- |
| O3HB 1u | -0.78 | -0.58 | -0.86 | -0.94 | -0.95 | -0.93 | -0.96 | -0.91 |
| O3HB 2u | -0.66 | -0.59 | -0.87 | -0.92 | -0.94 | -0.94 | -0.95 | -0.95 |
| O3HB 3u – 32u | -0.65 | -0.59 | -0.87 | -0.89 | -0.94 | -0.94 | -0.95 | -0.95 |
|  |  |  |  |  |  |  |  |  |
| O4HB 1u | -0.79 | -0.58 | -0.86 | -0.96 | -0.95 | -0.97 | -0.97 | -0.88 |
| O4HB 2u -32u | -0.63 | 0.60 | -0.87 | -0.93 | -0.93 | -0.94 | -0.96 | -0.93 |
|  |  |  |  |  |  |  |  |  |
| O3HV 1u | -0.78 | -0.58 | -0.86 | -0.89 | -0.91 | -0.89 | -0.94 | -0.88 |
| O3HV 2u | -0.66 | -0.59 | -0.87 | -0.89 | -0.91 | -0.90 | -0.92 | -0.94 |
| O3HV 3u – 32u | -0.65 | -0.59 | -0.87 | -0.88 | -0.92 | -0.90 | -0.92 | -0.94 |
|  |  |  |  |  |  |  |  |  |
| O4HV 1u | -0.75 | -0.58 | -0.86 | -0.98 | -0.96 | -0.96 | -0.97 | -0.61 |
| O4HV 2u | -0.61 | -0.59 | -0.87 | -0.94 | -0.94 | -0.97 | -0.96 | -0.83 |
| O4HV 3u – 32u | -0.61 | -0.59 | -0.87 | -0.91 | -0.93 | -0.96 | -0.96 | -0.83 |
|  |  |  |  |  |  |  |  |  |
| O3HVB | -0.66 | -0.59 | -0.87 | -0.90 | -0.91 | -0.91 | -0.94 | -0.91 |
| O3HBV | -0.67 | -0.59 | -0.87 | -0.88 | -0.90 | -0.89 | -0.92 | -0.90 |
| O3HVBV | -0.62 | -0.59 | -0.87 | -0.82 | -0.90 | -0.88 | -0.93 | -0.89 |
| O3HBVB | -0.62 | -0.59 | -0.87 | -0.87 | -0.91 | -0.91 | -0.94 | -0.91 |
| O3HVBVB | -0.60 | -0.59 | -0.87 | -0.87 | -0.91 | -0.91 | -0.94 | -0.91 |
| O3HBVBV | -0.61 | -0.59 | -0.87 | -0.82 | -0.90 | -0.88 | -0.93 | -0.89 |
|  |  |  |  |  |  |  |  |  |
| O4HBV | -0.61 | 0.59 | -0.88 | -0.86 | -0.92 | -0.96 | -0.95 | -0.81 |
| O4HVB | -0.55 | -0.59 | -0.87 | -0.92 | -0.91 | -0.94 | -0.95 | -0.80 |
| O4HBVB | -0.50 | 0.59 | -0.89 | -0.85 | -0.91 | -0.93 | -0.94 | -0.82 |
| O4HBVV | -0.59 | 0.59 | -0.89 | -0.83 | -0.92 | -0.97 | -0.95 | -0.85 |
| 04HVBV | -0.52 | -0.59 | -0.87 | -0.84 | -0.92 | -0.97 | -0.95 | -0.86 |
| O4HVBB | -0.54 | -0.59 | -0.87 | -0.95 | -0.92 | -0.92 | -0.95 | -0.86 |
