# Supplementary material for: In silico Assessment of Pharmacological Profile of Low Molecular Weight Oligo-Hydroxyalkanoates
Source: Front Bioeng Biotechnol. 2020 Nov 26;8:584010. doi: 10.3389/fbioe.2020.584010 (PMC7726197; doi:10.3389/fbioe.2020.584010)
Supplement: Supplementary file 2 [file Table_2.DOCX]

Supplementary Table 2. Probabilities of low molecular weight oligo-hydroxyalkanoates to produce toxicological endpoints predicted with the software admetSAR2.0: hERG – blockage of the potassium channel, EC – eye corrosion, EI – eye irritation, HEPT - hepatotoxicity. The predicted probabilities take values between 0 and 1 in the case of a biological activity that is present and between -1 and 0 when the activity is considered absent. Values are closer to 1 mean that biological effects are highly probable and values closer to -1 correspond to highly improbable biological effects. In this table u denotes the number of units in the oligomer, O3HB denotes the oligomer of 3HB, O3HV denotes the oligomer of 3HV, O4HB denote the oligomer of 4HB, O4HV denotes the oligomer of 4HV. In the case of co-oligomers, BV, VB, BVB, VBV, BVV, VBB, BVBV and VBVB illustrate the succession of the butyrate (B) and valerate (V) monomers in the oligomer chain

| **Oligomer** | **non hERG** | **non mutagen** | **non carcinogen** | **EC** | **EI** | **non HEPT** |
| --- | --- | --- | --- | --- | --- | --- |
| O3HB 1u | 0.83 | 0.95 | 0.76 | 0.84 | 0.98 | 0.95 |
| O3HB 2u | 0.86 | 0.89 | 0.74 | 0.71 | 0.96 | 0.80 |
| O3HB 3u | 0.86 | 0.89 | 0.74 | 0.65 | 0.61 | 0.70 |
| O3HB 4u | 0.82 | 0.89 | 0.74 | 0.65 | -0.67 | 0.60 |
| O3HB 5u | 0.80 | 0.89 | 0.74 | 0.65 | -0.83 | 0.68 |
| O3HB 6u | 0.73 | 0.89 | 0.74 | 0.65 | -0.87 | 0.55 |
| O3HB 7u | 0.66 | 0.89 | 0.74 | 0.65 | -0.89 | 0.55 |
| O3HB 8u | 0.61 | 0.89 | 0.74 | 0.65 | -0.90 | 0.55 |
| O3HB 16u – 32 u | 0.53 | 0.89 | 0.74 | 0.65 | -0.89 | 0.55 |
|  |  |  |  |  |  |  |
| O4HB 1u | 0.81 | 0.97 | 0.80 | 0.88 | 0.99 | 0.85 |
| O4HB 2u | 0.63 | 0.96 | 0.77 | 0.66 | 0.96 | 0.83 |
| O4HB 3u | 0.48 | 0.96 | 0.77 | 0.66 | 0.92 | 0.80 |
| O4HB 4u | 0.47 | 0.96 | 0.77 | 0.66 | 0.86 | 0.83 |
| O4HB 5u | 0.45 | 0.96 | 0.77 | 0.66 | 0.66 | 0.68 |
| O4HB u | 0.46 | 0.96 | 0.77 | 0.66 | -0.66 | 0.73 |
| O4HB 7u | 0.48 | 0.96 | 0.77 | 0.66 | -0.74 | 0.75 |
| O4HB 8u | 0.45 | 0.96 | 0.77 | 0.66 | -0.77 | 0.70 |
| O4HB 16u- 32 u | 0.42 | 0.96 | 0.77 | 0.66 | -0.84 | 0.68 |
|  |  |  |  |  |  |  |
| O3HV 1u | 0.77 | 0.89 | 0.76 | 0.65 | 0.96 | 0.85 |
| O3HV 2u | 0.75 | 0.79 | 0.74 | 0.57 | 0.86 | 0.80 |
| O3HV 3u | 0.75 | 0.78 | 0.74 | 0.51 | -0.51 | 0.75 |
| O3HV 4u | 0.69 | 0.78 | 0.74 | 0.51 | -0.69 | 0.65 |
| O3HV 8u | 0.51 | 0.78 | 0.74 | 0.51 | -0.89 | 0.63 |
| O3HV 16u – 20u | 0.44 | 0.78 | 0.74 | 0.51 | -0.89 | 0.60 |
|  |  |  |  |  |  |  |
| O4HV 1u | 0.85 | 0.98 | 0.76 | 0.96 | 0.90 | 0.68 |
| O4HV 2u | 0.70 | 0.88 | 0.74 | 0.57 | -0.74 | 0.75 |
| O4HV 3u | 0.68 | 0.88 | 0.74 | 0.48 | -0.82 | 0.80 |
| O4HV 4u | 0.65 | 0.88 | 0.74 | 0.48 | -0.85 | 0.60 |
| O4HV 6u | 0.63 | 0.88 | 0.74 | 0.48 | -0.89 | 0.63 |
| O4HV 8u | 0.55 | 0.88 | 0.74 | 0.48 | -0.90 | 0.60 |
| O4HV 12u – 32 u | 0.45 | 0.88 | 0.74 | 0.48 | -0.90 | 0.58 |
|  |  |  |  |  |  |  |
| O3HVB | 0.86 | 0.88 | 0.74 | 0.53 | 0.83 | 0.78 |
| O3HBV | 0.77 | 0.83 | 0.74 | 0.50 | 0.83 | 0.73 |
| O3HVBV | 0.82 | 0.76 | 0.74 | -0.63 | 0.53 | 0.75 |
| O3HBVB | 0.83 | 0.79 | 0.74 | 0.46 | -0.61 | 0.70 |
| O3HVBVB | 0.78 | 0.79 | 0.74 | 0.46 | -0.73 | 0.63 |
| O3HBVBV | 0.68 | 0.76 | 0.74 | -0.63 | -0.72 | 0.60 |
|  |  |  |  |  |  |  |
| O4HBV | 0.69 | 0.92 | 0.70 | -0.55 | -0.81 | 0.78 |
| O4HVB | 0.73 | 0.96 | 0.74 | -0.66 | -0.68 | 0.82 |
| O4HBVB | 0.52 | 0.91 | 0.70 | -0.88 | -0.68 | 0.83 |
| O4HBVV | 0.45 | 0.89 | 0.70 | -0.80 | -0.79 | 0.78 |
| 04HVBV | 0.47 | 0.90 | 0.74 | -0.74 | -0.68 | 0.78 |
| O4HVBB | 0.63 | 0.96 | 0.74 | -0.60 | -0.55 | 0.88 |
