# Supplementary material for: In silico Assessment of Pharmacological Profile of Low Molecular Weight Oligo-Hydroxyalkanoates
Source: Front Bioeng Biotechnol. 2020 Nov 26;8:584010. doi: 10.3389/fbioe.2020.584010 (PMC7726197; doi:10.3389/fbioe.2020.584010)
Supplement: Supplementary file 3 [file Table_3.docx]

Supplementary Table 3

Predictions obtained using Pred-Skin 3.0 software regarding the skin sensitization potential of the investigated oligomers. There are five models that are used in these predictions: DPRA - Direct Peptide Reactivity Assay, KeratinoSens sensitization of human keratinocytes test, H-CLAT - human Cell Line Activation Test, LLNA - murine local lymph node assay, HRIPT/HMT - human repeated insult patch test and human maximization test. There also is a Bayesian consensus model that is generated by averaging the predictions of individual models. In this table u denotes the number of units in the oligomer, O3HB denotes the oligomer of 3HB, O3HV denotes the oligomer of 3HV, O4HB denote the oligomer of 4HB, O4HV denotes the oligomer of 4HV. In the case of co-oligomers, BV, VB, BVB, VBV, BVV, VBB, BVBV and respectively VBVB illustrate the succession of the 3-hydroxybutyrate /4-hydroxybutyrate (B) and 3-hydroxyvalerate/4-hydroxyvalerate (V) monomers in the co-oligomeric chain, respectively.

| **Oligomer** | **Prediction DPRA** | **Prediction** | **Prediction h-CLAT** | **Prediction LLNA** | **Prediction HRIPT/HMT** | **Bayesian Outcome** |
| --- | --- | --- | --- | --- | --- | --- |
| O3HB 1u | Non-Sensitizer  (Confiability 92.9%) | Non-Sensitizer  (Confiability 92.0%) | Non-Sensitizer  (Confiability 62.0%) | Non-Sensitizer  (Confiability 99.9%) | Non-Sensitizer  (Confiability 99.2%) | Non-sensitizer  (Confiability High) |
| O3HB 2u | Non-Sensitizer  (Confiability 87.6%) | Sensitizer  (Confiability 89.8%) | Non-Sensitizer  (Confiability 62.0%) | Non-Sensitizer  (Confiability 99.7%) | Non-Sensitizer  (Confiability 95.6%) | Non-sensitizer  (Confiability High) |
| O3HB 3u | Non-Sensitizer  (Confiability 78.6%) | Sensitizer  (Confiability 97.9%) | Non-Sensitizer  (Confiability 59.7%) | Non-Sensitizer  (Confiability 99.8%) | Non-Sensitizer  (Confiability 87.0%) | Non-sensitizer  (Confiability High) |
| O3HB 4u | Non-Sensitizer  (Confiability 67.4%) | Sensitizer  (Confiability 97.8%) | Non-Sensitizer  (Confiability 59.7%) | Non-Sensitizer  (Confiability 99.9%) | Non-Sensitizer  (Confiability 78.5%) | Non-sensitizer  (Confiability High) |
| O3HB 5u – 8u | Non-Sensitizer  (Confiability 71.0%) | Sensitizer  (Confiability 96.8%) | Non-Sensitizer  (Confiability 59.7%) | Non-Sensitizer  (Confiability 99.9%) | Non-Sensitizer  (Confiability 76.3%) | Non-sensitizer  (Confiability High) |
| O3HB 16u – 32u | Too big to be computed | | | | | |
| O4HB 1u | Non-Sensitizer  (Confiability 88.1%) | Non-Sensitizer  (Confiability 92.7%) | Non-Sensitizer  (Confiability  60.8%) | Non-Sensitizer  (Confiability 99.9%) | Non-Sensitizer  (Confiability 80.8%) | Non-sensitizer  (Confiability High) |
| O4HB 2u | Non-Sensitizer  (Confiability 57.6%) | Sensitizer  (Confiability 84.2%) | Non-Sensitizer  (Confiability 65.0%) | Non-Sensitizer  (Confiability 100.0%) | Non-Sensitizer  (Confiability 81.7%) | Non-sensitizer  (Confiability High) |
| O4HB 3u | Non-Sensitizer  (Confiability 82.2%) | Sensitizer  (Confiability 70.8%) | Non-Sensitizer  (Confiability 65.0%) | Non-Sensitizer  (Confiability 100.0%) | Non-Sensitizer  (Confiability 84.2%) | Non-sensitizer  (Confiability High) |
| O4HB 4u – 20u | Non-Sensitizer  (Confiability 88.9%) | Sensitizer  (Confiability 73.7%) | Non-Sensitizer  (Confiability 65.0%) | Non-Sensitizer  (Confiability 100.0%) | Non-Sensitizer  (Confiability 85.3%) | Non-sensitizer  (Confiability High) |
| O4HB 24u – 32u | Too big to be computed | | | | | |
| O3HV 1u | Non-Sensitizer  (Confiability 54.3%) | Sensitizer  (Confiability 56.1%) | Non-Sensitizer  (Confiability 66.0%) | Non-Sensitizer  (Confiability 99.9%) | Non-Sensitizer  (Confiability 97.6%) | Non-sensitizer  (Confiability High) |
| O3HV 2u | Non-Sensitizer  (Confiability 88.2%) | Non-Sensitizer  (Confiability 84.9%) | Non-Sensitizer  (Confiability 63.7%) | Non-Sensitizer  (Confiability 100.0%) | Non-Sensitizer  (Confiability 97.6%) | Non-sensitizer  (Confiability High) |
| O3HV 3u | Non-Sensitizer  (Confiability 82.6%) | Non-Sensitizer  (Confiability 89.6%) | Non-Sensitizer  (Confiability 59.7%) | Non-Sensitizer  (Confiability 100.0%) | Non-Sensitizer  (Confiability 96.1%) | Non-sensitizer  (Confiability High) |
| O3HV 4u | Sensitizer  (Confiability 65.6%) | Non-Sensitizer  (Confiability 77.8%) | Non-Sensitizer  (Confiability 59.7%) | Non-Sensitizer  (Confiability 100.0%) | Non-Sensitizer  (Confiability 92.4%) | Non-sensitizer  (Confiability High) |
| O3HV 8u | Sensitizer  (Confiability 64.7%) | Non-Sensitizer  (Confiability 79.4%) | Non-Sensitizer  (Confiability 59.7%) | Non-Sensitizer  (Confiability 100.0%) | Non-Sensitizer  (Confiability 93.3%) | Non-sensitizer  (Confiability High) |
| O3HV 16u- 32u | Too big to be computed | | | | | |
| O4HV 1u | Non-Sensitizer  (Confiability 79.6%) | Non-Sensitizer  (Confiability 93.7%) | Non-Sensitizer  (Confiability 59.7%) | Non-Sensitizer  (Confiability 99.9%) | Non-Sensitizer (-  (Confiability 98.7%) | Non-sensitizer  (ConfiabilityHigh) |
| O4HV 2u | Non-Sensitizer  (Confiability 78.4%) | Sensitizer  (Confiability 63.7%) | Non-Sensitizer  (Confiability 58.9%) | Non-Sensitizer  (Confiability 99.8%) | Non-Sensitizer  (Confiability 97.4%) | Non-sensitizer  (Confiability High) |
| O4HV 3u | Non-Sensitizer  (Confiability 70.4%) | Sensitizer  (Confiability 96.8%) | Non-Sensitizer  (Confiability 57.3%) | Non-Sensitizer  (Confiability 99.8%) | Non-Sensitizer  (Confiability 95.7%) | Non-sensitizer  (Confiability High) |
| O4HV 4u – 16u | Non-Sensitizer (Confiability 52.5%) | Sensitizer  (Confiability 97.2%) | Non-Sensitizer  (Confiability 57.3%) | Non-Sensitizer  (Confiability 99.8%) | Non-Sensitizer  (Confiability 95.7%) | Non-sensitizer  (Confiability High) |
| O4HV 20U - 32U | Too big to be computed | | | | | |
| O3HVB | Non-Sensitizer  (Confiability 81.8%) | Sensitizer  (Confiability 75.3%) | Non-Sensitizer  (Confiability 61.6%) | Non-Sensitizer  (Confiability 99.9%) | Non-Sensitizer  (Confiability 88.8%) | Non-sensitizer  (Confiability High) |
| O3HBV | Non-Sensitizer  (Confiability 88.2%) | Non-Sensitizer  (Confiability 70.5%) | Non-Sensitizer  (Confiability 62.1%) | Non-Sensitizer  (Confiability 100.0%) | Non-Sensitizer  (Confiability 98.2%) | Non-sensitizer  (Confiability High) |
| O3HVBV | Non-Sensitizer  (Confiability 59.7%) | Sensitizer  (Confiability 95.0%) | Non-Sensitizer  (Confiability 58.4%) | Non-Sensitizer  (Confiability 100.0%) | Non-Sensitizer  (Confiability 54.4%) | Non-sensitizer  (Confiability High) |
| O3HBVB | Sensitizer  (Confiability 70.8%) | Sensitizer  (Confiability 94.8%) | Non-Sensitizer  (Confiability 57.9%) | Non-Sensitizer  (Confiability 99.9%) | Non-Sensitizer  (Confiability 89.9%) | Non-sensitizer  (Confiability High) |
| O3HVBVB | Sensitizer  (Confiability 83.0%) | Non-Sensitizer  (Confiability 65.8%) | Non-Sensitizer  (Confiability 57.9%) | Non-Sensitizer  (Confiability 100.0%) | Non-Sensitizer  (Confiability 92.7%) | Non-sensitizer  (Confiability High) |
| O3HBVBV | Non-Sensitizer  (Confiability 80.4%) | Sensitizer  (Confiability 94.6%) | Non-Sensitizer  (Confiability 58.4%) | Non-Sensitizer  (Confiability 100.0%) | Sensitizer  (Confiability 73.4%) | Sensitizer  (Confiability High) |
| O4HBV | Non-Sensitizer  (Confiability 93.3%) | Non-Sensitizer  (Confiability 79.2%) | Non-Sensitizer  (Confiability 61.2%) | Non-Sensitizer  (Confiability 99.9%) | Non-Sensitizer  (Confiability 97.5%) | Non-sensitizer  (Confiability High) |
| O4HVB | Non-Sensitizer  (Confiability 92.9%) | Non-Sensitizer  (Confiability 94.7%) | Non-Sensitizer  (Confiability 62.0%) | Non-Sensitizer  (Confiability 00.0%) | Sensitizer  (Confiability 66.9%) | Sensitizer  (Confiability High) |
| O4HBVB | Non-Sensitizer  (Confiability 96.1%) | Non-Sensitizer  (Confiability 80.9%) | Non-Sensitizer  (Confiability 64.5%) | Non-Sensitizer  (Confiability 99.9%) | Non-Sensitizer  (Confiability 87.6%) | Non-sensitizer  (Confiability High) |
| O4HBVV | Non-Sensitizer  (Confiability 88.7%) | Sensitizer  (Confiability 96.6%) | Non-Sensitizer  (Confiability 60.3%) | Non-Sensitizer  (Confiability 99.9%) | Non-Sensitizer  (Confiability  97.0%) | Non-sensitizer  (Confiability High) |
| O4HVBV | Non-Sensitizer  (Confiability 92.7%) | Non-Sensitizer  (Confiability 90.0%) | Non-Sensitizer  (Confiability  65.2%) | Non-Sensitizer  (Confiability 100.0%) | Non-Sensitizer  (Confiability 86.8%) | Non-sensitizer  (Confiability High) |
| O4HVBB | Non-Sensitizer  (Confiability 88.5%) | Sensitizer  (Confiability 87.8%) | Non-Sensitizer  (Confiability 64.8%) | Non-Sensitizer  (Confiability 100.0%) | Sensitizer  (Confiability 67.8%) | Sensitizer  (Confiability High) |
