# Supplementary material for: In silico Assessment of Pharmacological Profile of Low Molecular Weight Oligo-Hydroxyalkanoates
Source: Front Bioeng Biotechnol. 2020 Nov 26;8:584010. doi: 10.3389/fbioe.2020.584010 (PMC7726197; doi:10.3389/fbioe.2020.584010)
Supplement: Supplementary file 4 [file Table_4.docx]

Supplementary Table 4. Predictions concerning carcinogenic and mutagenic potential of low molecular weight OHAs obtained using Toxtree & CarcinoPred-EL computational tools. In this table u denotes the number of units in the oligomer, O3HB denotes the oligomer of 3HB, O3HV denotes the oligomer of 3HV, O4HB denote the oligomer of 4HB, O4HV denotes the oligomer of 4HV. In the case of co-oligomers, BV, VB, BVB, VBV, BVV, VBB, BVBV and respectively VBVB illustrate the succession of the butyrate (B) and respectively valerate (V) monomers in the oligomer chain.

| **oligomer** | **Carcinogenity** | | | **Ames toxicity**  **(mutagenicity)** |
| --- | --- | --- | --- | --- |
|  | **Toxtree** | | **CarcinoPred-EL** | **Toxtree** |
|  | **Non-genotoxic carcinogenicity** | **Genotoxic carcinogenicity** | **carcinogen** | **Ames toxic** |
| O3HB 1u -32 u | No | No | No | No |
|  |  |  |  |  |
| O3HV 1u – 32u | No | No | No | No |
|  |  |  |  |  |
| O4HB 1u – 32u | No | No | No | No |
|  |  |  |  |  |
| O4HV 1u- 32u | No | No | No | No |
|  |  |  |  |  |
| O3HVB | No | No | No | No |
| O3HBV | No | No | No | No |
| O3HVBV | No | No | No | No |
| O3HBVB | No | No | No | No |
| O3HVBVB | No | No | No | No |
| O3HBVBV | No | No | No | No |
|  |  |  |  |  |
| O4HBV | No | No | No | No |
| O4HVB | No | No | No | No |
| O4HBVB | No | No | No | No |
| O4HBVV | No | No | No | No |
| 04HVBV | No | No | No | No |
| O4HVBB | No | No | No | No |
