# Supplementary material for: In silico Assessment of Pharmacological Profile of Low Molecular Weight Oligo-Hydroxyalkanoates
Source: Front Bioeng Biotechnol. 2020 Nov 26;8:584010. doi: 10.3389/fbioe.2020.584010 (PMC7726197; doi:10.3389/fbioe.2020.584010)
Supplement: Supplementary file 5 [file Table_5.docx]

Supplementary Table 5. Predictions concerning the potential binding of investigated low molecular weight OHAs to the human nuclear receptors obtained using Endocrine Disruptome computational tool: Androgen receptor (AR), estrogen receptors α (ERα) and β (ERβ), glucocorticoid receptor (GR), liver X receptors α (LXRα) and β (LRXβ), peroxisome proliferator activated receptors α (PPRAα), β/δ (PPRAβ) and γ (PPRAγ), retinoid X receptor α (RXRα), thyroid receptors α (TRα), β (TRβ), and “an” means antagonistic effect. White cells correspond to compounds reflecting a low probability of interacting with the indicated nuclear receptor and grey cells correspond to compounds affecting the nuclear receptors. In this table u denotes the number of units in the oligomer, O3HB denotes the oligomer of 3HB, O3HV denotes the oligomer of 3HV, O4HB denote the oligomer of 4HB, O4HV denotes the oligomer of 4HV. In the case of co-oligomers, BV, VB, BVB, VBV, BVV, VBB, BVBV and respectively VBVB illustrate the succession of the 3-hydroxybutyrate /4-hydroxybutyrate (B) and 3-hydroxyvalerate/4-hydroxyvalerate (V) monomers in the co-oligomeric chain, respectively. O3HBs, O3HVs, O4HBs, and O4HVs containing more than 8 monomeric units were found to be too big to accommodate in the binding sites of the nuclear receptors.

| **oligomer** | **AR** | **Ar an** | **ERα** | **ERα an** | **ERβ** | **ERβ an** | **GR** | **GR an** | **LXRα** | **LXRβ** | **PPARα** | **PPARβ** | **PPARγ** | **RXRα** | **TRα** | **TRβ** |
| --- | --- | --- | --- | --- | --- | --- | --- | --- | --- | --- | --- | --- | --- | --- | --- | --- |
| O3HB 1u – 5u |  |  |  |  |  |  |  |  |  |  |  |  |  |  |  |  |
| O3HB 6u – 8u |  |  |  |  |  |  |  |  |  |  |  |  |  |  |  |  |
| O4HB 1u – 8u |  |  |  |  |  |  |  |  |  |  |  |  |  |  |  |  |
| O3HV 1u-3u |  |  |  |  |  |  |  |  |  |  |  |  |  |  |  |  |
| O3HV 4u-8u |  |  |  |  |  |  |  |  |  |  |  |  |  |  |  |  |
| O4HV 1u-5u |  |  |  |  |  |  |  |  |  |  |  |  |  |  |  |  |
| O4HV 6u-8u |  |  |  |  |  |  |  |  |  |  |  |  |  |  |  |  |
| O3HVB |  |  |  |  |  |  |  |  |  |  |  |  |  |  |  |  |
| O3HBV |  |  |  |  |  |  |  |  |  |  |  |  |  |  |  |  |
| O3HVBV |  |  |  |  |  |  |  |  |  |  |  |  |  |  |  |  |
| O3HBVB |  |  |  |  |  |  |  |  |  |  |  |  |  |  |  |  |
| O3HVBVB |  |  |  |  |  |  |  |  |  |  |  |  |  |  |  |  |
| O3HBVBV |  |  |  |  |  |  |  |  |  |  |  |  |  |  |  |  |
| O4HBV |  |  |  |  |  |  |  |  |  |  |  |  |  |  |  |  |
| O4HVB |  |  |  |  |  |  |  |  |  |  |  |  |  |  |  |  |
| O4HBVB |  |  |  |  |  |  |  |  |  |  |  |  |  |  |  |  |
| O4HBVV |  |  |  |  |  |  |  |  |  |  |  |  |  |  |  |  |
| 04HVBV |  |  |  |  |  |  |  |  |  |  |  |  |  |  |  |  |
| O4HVBB |  |  |  |  |  |  |  |  |  |  |  |  |  |  |  |  |
